# Effect of Long-Term Supplementation With Silkworm Pupae Oil on the Methane Yield, Ruminal Protozoa, and Archaea Community in Sheep

**DOI:** 10.3389/fmicb.2022.780073

**Published:** 2022-03-08

**Authors:** Govindasamy Thirumalaisamy, Pradeep Kumar Malik, Shraddha Trivedi, Atul Purushottam Kolte, Raghavendra Bhatta

**Affiliations:** ^1^ICAR-National Institute of Animal Nutrition and Physiology, Bengaluru, India; ^2^ICAR-National Dairy Research Institute, Karnal, India

**Keywords:** archaea, long-term feeding, methane yield, sheep, silkworm pupae oil

## Abstract

Supplementation with lipids and oils is one of the most efficient strategies for reducing enteric methane emission. However, high costs and adverse impacts on fiber degradation restrict the use of conventional oils. Silkworm pupae, a non-conventional oil source rarely used for human consumption in India, could be one of the cheaper alternatives for methane mitigation. The objective of this study was to investigate the effect on sheep of long-term supplementation (180 days) of silkworm pupae oil (SWPO) with two distinct supplementation regimes (daily and biweekly) on daily enteric methane emission, methane yield, nutrient digestibility, rumen fermentation, ruminal archaea community composition, and protozoal population. The effect of the discontinuation of oil supplementation on enteric methane emission was also investigated. Eighteen adult male sheep, randomly divided into three groups (*n* = 6), were provisioned with a mixed diet consisting of 10.1% crude protein (CP) and 11.7 MJ/kg metabolizable energy formulated using finger millet straw and concentrate in a 55:45 ratio. SWPO was supplemented at 2% of dry matter intake (DMI) in test groups either daily (CON) or biweekly (INT), while no oil was supplemented in the control group (CTR). DMI (*p* = 0.15) and CP (*p* = 0.16) in the CON and INT groups were similar to that of the CTR group; however, the energy intake (MJ/kg) in the supplemented groups (CON and INT) was higher (*p* < 0.001) than in CTR. In the CON group, body weight gain (kg, *p* = 0.02) and average daily gain (g, *p* = 0.02) were both higher than in the CTR. The daily methane emission in the CON (17.5 g/day) and INT (18.0 g/day) groups was lower (*p* = 0.01) than the CTR group (23.6 g/day), indicating a reduction of 23–25% due to SWPO supplementation. Similarly, compared with the CTR group, methane yields (g/kg DMI) in test groups were also significantly lower (*p* < 0.01). The transient nature of the anti-methanogenic effect of SWPO was demonstrated in the oil discontinuation study, where daily methane emission reverted to pre-supplementation levels after a short period. The recorded methanogens were affiliated to the families *Methanobacteriaceae*, *Methanomassilliicoccaceae*, and *Methanosarcinaceae*. The long-term supplementation of oil did not induce any significant change in the rumen archaeal community, whereas minor species such as *Group3b* exhibited differing abundance among the groups. *Methanobrevibacter*, irrespective of treatment, was the largest genus, while *Methanobrevibacter gottschalkii* was the dominant species. Oil supplementation in CON and INT compared with CTR decreased (*p* < 0.01) the numbers of total protozoa (× 10^7^ cells/ml), *Entodiniomorphs* (× 10^7^ cells/ml), and *Holotrichs* (× 10^6^ cells/ml). SWPO continuous supplementation (CON group) resulted in the largest reduction in enteric methane emission and relatively higher body weight gain (*p* = 0.02) in sheep.

## Introduction

The global livestock sector accounts for 14.5% of anthropogenic greenhouse gas emissions (Gerber et al., 2013). Enteric fermentation alone is accountable for 87–97 Tg of methane produced on an annual basis (Chang et al., 2019). Apart from contributing to global warming, the enteric methane emissions from livestock are also responsible for 2–12% of dietary energy loss (Johnson and Johnson, 1995), where every liter of methane carries 39.5 kJ of energy from the host animal (Guan et al., 2006).

The rumen is an ideal habitat for protozoa, where they live in close association with prokaryotic microbes (Newbold et al., 2015). After bacteria, protozoa are the second most abundant microbes, constituting up to 50% of the rumen biomass (Newbold et al., 2015) with the overall dominance of ciliated protozoa (Morgavi et al., 2010). Rumen protozoa are not essential for animal survival, yet they perform important functions such as protein breakdown, bacterial predation (Williams and Coleman, 1992), reduction of the shedding of potential pathogens (Newbold et al., 2015), lipid metabolism, and shifts in volatile fatty acids production (Eugène et al., 2004). However, their presence in the rumen negatively impacts the energy efficiency of the rumen ecosystem (Newbold et al., 2015). One of the major functions of rumen protozoa is to transfer hydrogen to other microbes, particularly to the methanogens (Li et al., 2018). Methanogens belong to the phylum *Euryarchaeota* (Balch et al., 1979), constituting 3–4% of the rumen microbiota (Yanagita et al., 2000). Rumen protozoa associated methanogens are responsible for 37% of enteric methane emissions (Machmüller et al., 2003). It has been reported that the counts of rumen protozoa are linearly related to methane emissions; however, methanogenesis is also regulated by other mechanisms independent of protozoa (Guyader et al., 2014).

Oil supplementation is a promising method for increasing diet density. In addition, supplementation with lipids effectively reduces enteric methane production (Morgavi et al., 2010). Oil supplementation is considered a promising approach for enteric methane mitigation in ruminants through a shift in rumen fermentation or microbiota composition (Vargas et al., 2020). Commonly used edible oils such as linseed, rapeseed, palm, and canola are expensive (Government of India, 2021). Silkworm pupae oil (SWPO) is non-conventional, inexpensive, and adequately available in India (Srinivas et al., 2019). The oil contains reasonably good combinations of both unsaturated and saturated fatty acids (Thirumalaisamy et al., 2020). Polyunsaturated fatty acids reduce the abundance of protozoa and, therefore, can be one of the potential mitigation options for reducing enteric methane emissions (Guyader et al., 2017).

Recently, the supplementation of SWPO in a short-term study decreased the enteric methane emission of sheep (Thirumalaisamy et al., 2020). Recently, Jayanegara et al. (2020) evaluated oils from five different insect sources for their impact on *in vitro* methane and concluded that mealworm and cricket oil supplementation at 5% effectively decreased methane production by 26–33%.

Due to adaptability of the rumen ecosystem, the methane inhibition is usually short-lived (Mathison et al., 1998), and animals return to normal methane emissions after withdrawal of ameliorating supplement from the diet (Hristov et al., 2013). Although this is a known issue, nevertheless, very few studies were undertaken to evaluate the impact of long-term supplementation on methane emission. Therefore, studies are needed to establish the persistency of methane mitigation in the long term (Beauchemin et al., 2020). Previous research (Thirumalaisamy et al., 2020) has shown that the short-term supplementation of SWPO significantly reduces enteric methane emission, which encouraged us to evaluate the effect over the long term. We hypothesized that long-term supplementation of SWPO may decrease methane emissions and methane yield by altering the archaeal community composition and reducing ruminal protozoa. Therefore, the current study was designed to investigate (1) the effects of long-term supplementation of SWPO in two distinct supplementation regimes (daily and biweekly) on daily enteric methane emission, methane yield (g/kg dry matter intake), nutrient digestibility, rumen fermentation, ruminal archaea diversity, and protozoal numbers in sheep, and (2) whether discontinuation of oil supplementation affects methane emission.

## Materials and Methods

### Animals and Feeding

The animal study was approved by the Committee for Control and Supervision of Experiments on Animals (CPCSEA), Ministry of Fisheries, Animal Husbandry, and Dairying, Government of India (approval no. 25/14/2017-CPCSEA). All procedures were carried out as per the guidelines of the Institute Animal Ethics Committee.

A 180-day-long animal study was carried out in (*n* = 18) 18-month-old male *Mandya* sheep (BW 24.1 ± 0.27 kg), randomly divided into three groups of six. The animals were housed in a well-ventilated shed constructed with half cemented walls and half iron mesh. The iron mesh was fixed into the cemented walls at 1.8 m above the ground on the east and west sides. The sheep were housed in individual pens having free access to clean drinking water and feed throughout the day. Initially, all the animals were dewormed with anthelminthic fenbendazole at 5 mg/kg BW. The animals were offered a total mixed ration (TMR) formulated with 55% finger millet straw (*Eleusine coracana*) and 45% concentrate mixture. The TMR was formulated to meet nutrient requirement as per ICAR (2013) feeding standards. The concentrate mixture was prepared using maize grain (320 g/kg), wheat bran (400 g/kg), soybean meal (130 g/kg), groundnut cake (120 g/kg), mineral mixture (20 g/kg), and salt (10 g/kg). The TMR offered to the animals in different groups was similar in all nutritional aspects and contained 10.1% crude protein (CP) and 11.7 MJ/kg metabolizable energy (ME). The TMR was offered *ad libitum* twice a day at 09:00 and 14:00, and clean drinking water was accessible throughout the day.

The oil extracted from dried silkworm pupae using the solvent extraction method (*n*-hexane) was purchased from a local vendor in the suburb of Bangalore and stored in an airtight container. The SWPO was filled in a plastic container (2 L; NJ Phillips, Australia) connected to an automatic oral drenching gun (NJ Phillips, part no SH46) for administering the required volume. The oil was supplemented at 2% of dry matter intake either daily (CON) or biweekly (INT), whereas animals in the control group (CTR) were offered TMR without oil. Thus, the animals in the INT group received oil daily for 1 week and then this was discontinued for the subsequent week.

Dry matter intake (DMI, g/day) was recorded at monthly interval for three consecutive days throughout the experiment to adjust SWPO volume (w/v) in the CON and INT groups.

### Chemical Analysis

The chemical composition of the feed ingredients, TMR, refusals, and feces were analyzed in triplicate. The dry matter content in the samples was determined as per AOAC (2012) in a hot air oven at 100°C for 12 h, and dried samples were ground using a Cyclotec mill. For determining total ash, the samples were initially burnt in crucibles on a hot plate and later transferred to a muffle furnace at 550°C for 4 h (AOAC, 2012). Organic matter (OM) was determined by subtracting the total ash from the initial dry weight of the sample and expressed as a percentage. Crude protein (CP, N × 6.25) was determined as per AOAC (2005) using an automatic nitrogen analyzer (Gerhardt, Germany). The crude fiber (CF) and fiber fractions such as neutral detergent fiber (NDF) and acid detergent fiber (ADF) were determined using an automatic fiber analyzer (Fibretherm FT12, Gerhardt, Germany) in accordance with AOAC (2005) and Van Soest et al. (1991). The ether extract (EE) was estimated using Soxtherm (Gerhardt, Germany) as per the standard method (AOAC, 2005). The gross energy content (kcal/100 g DM) of TMR and feed ingredients was calculated following the equation of Crisan and Sands (1978), and expressed as metabolizable energy (MJ/kg DM) after considering digestible and urinary losses. The chemical composition (g/kg DM) is presented in Table 1.

**TABLE 1:** Chemical composition (g/kg DM) of feed ingredients and TMR.

Attributes	Ingredients/diet						
	Finger millet straw	Concentrate	Maize	Soybean meal	Groundnut cake	Wheat bran	TMR
OM	910	942	982	914	942	982	928
CP	36.5	193	91.5	447	424	141	101
EE	10.3	32.2	47.4	101	57.9	47.4	20.1
NDF	670	460	514	498	354	514	618
ADF	508	131	93.1	262	224	93.1	357
CF	330	43.9	26.8	94.1	106	268	255
TA	89.7	58.0	18.3	85.8	57.7	18.3	71.7
NFE	429	579	711	266	262	711	452
ME* (MJ/kg)	11.6	11.6	12.8	9.50	10.7	12.8	11.7

OM, organic matter; CP, crude protein (N_2_ × 6.25); EE, ether extract; NDF, neutral detergent fiber; ADF, acid detergent fiber; CF, crude fiber; TA, total ash; TMR, total mixed ration. Nitrogen-free extract (NFE) was calculated by subtracting the %CP + %EE + %CF + %TA + %moisture from 100. ME is the metabolizable energy, calculated from the gross energy of feed by multiplying by 0.82. *The energy value of TMR plus oil was 12.24 MJ.

Ammonia-N concentration in the ruminal fluid samples was determined as per Conway (1957) following the microdiffusion technique. The microdiffusion cell has two chambers—1 ml of the mixed boric acid (2%) indicator (methyl red 66 mg, methylene blue 33 mg in 100 ml alcohol) was pipetted into the inner chamber while an equal volume of saturated sodium carbonate was placed in the outer chamber of the disc. A measure of 1 ml of strained ruminal fluid was pipetted into the outer chamber directly opposite to the sodium carbonate, and then the microdiffusion cell was immediately covered with the lid. To allow the mixing of the contents of the outer chamber, the disc was gently rotated and incubated for 2 h at room temperature. After the incubation, the contents of the inner chamber were titrated against 0.01 N sulfuric acid until the color turned pink. Ammonia-N was determined using the following formula:


$$\text{Ammonia}-\text{N}\,\left(\text{mg}/\text{dl}\right)=\text{ml}\,\text{of}\,0.001\,\text{N}\,H_{2}\,S\,O_{4}\times 1$$

Volatile fatty acids (VFA) concentration in the ruminal fluid samples was determined according to Filípek and Dvoøák (2009) using a gas chromatograph (Agilent 7890B, Santa Clara, United States). In brief, preserved metaphosphoric acid mixed supernatant samples were thawed at room temperature, and after a short spinning at 13,000 rpm for 5 min, about 1.5 ml of the sample was transferred to GC screw vials (2 ml; Agilent Technologies). The screw vials containing fluid samples and the VFA standard and washing solvent were placed in the automatic injector port (G4513A). The injector dispensed a fixed volume (1 μl) of the sample while maintaining the 20:1 splitting ratio. The injector was equipped with a glass liner containing glass wool to separate dirt particles from the sample. Nitrogen gas (N_2_) at a flow rate of 2 ml per min was used as a carrier gas. The gas chromatograph was equipped with the FFAP column (CP7485, 25 m × 0.32 mm × 0.30 μm; Agilent Technologies) and flame ionization detector (FID). The following conditions were maintained during the analysis: temperature program, 59–250°C (20°C/min, 10 min); injector temperature, 230°C; detector temperature, 280°C. The analysis time was approximately 16.7 min. The concentration of individual volatile fatty acids was determined using the following formula:


$$\text{VFA con}.\;(\text{mmol})\;\;=\;\frac{\text{Peak area of sample }\times \;\text{Conc}.\text{ of standard }\times \;\text{dilution}}{\text{Peak area of standard}}$$

Total metabolic hydrogen produced was calculated using the concentration of volatile fatty acids as per the equation of Marty and Demeyer (1973) and hydrogen utilized (%) in methane was calculated based on daily enteric methane emission.


Total⁢2⁢H⁢produced=2⁢A+P+4⁢B+3⁢V

### Body Weight, Nutrient Digestibility, and Methane Measurement

During the entire experimental period (180 days), before morning feeding the animals were weighed at monthly intervals on a digital balance (Blue Bird, 300 kg). Their total weight gain (kg) was calculated as the difference between final and initial live-weight, and average daily gain (ADG, g/day) was calculated by dividing total weight gain by the number of experimental days.

A digestibility trial was conducted for 10 days between days 171 and 180, and apparent nutrient digestibility was determined. In brief, the quantity of daily TMR offered, feed refusals, and feces were recorded and the representative samples were collected from the individual animal. The DMI (g/day) of the individual animal was calculated by subtracting the quantity of dry refusals from the feed offered. The energy intake was calculated by considering daily DMI (g/day) and energy content of TMR (ME, MJ/kg DM), as described under chemical analysis. The digestibility coefficient of various nutrients was determined by using the following equation:


$$\mathrm{Digestibility}\,\mathrm{coefficient}=\frac{\mathrm{Nutrient}\,\mathrm{intake}-\mathrm{Excretion}\,\mathrm{of}\,\mathrm{nutrien}\,\text{t}}{\mathrm{Intake}\,\mathrm{of}\,\mathrm{nutrient}}$$

The sulfur hexafluoride (SF_6_) tracer technique (Berndt et al., 2014) was employed for the quantification of daily enteric methane emission for 10 consecutive days (days 171–180). During methane measurements, animals in the INT group received SWPO from days 171 to 175, whereas the remaining 5 days (176–180) were under the supplementation break. The brass permeation tubes (8.5 mm diameter, 34 mm long, 4.8 mm wide bore, and 30 mm deep with blind hole) fitted with a Swagelok nut (7 mm diameter) were used as a source of SF_6_. A Teflon septum (0.24 mm PTFE) was placed below the SS frit (3/8″ OD, 2 μm pore size), and the tubes were closed. The permeation tubes were charged with 750 ± 48.18 mg SF_6_ (99.9%) in liquid nitrogen and retained in an incubator at 39°C for 70 days. The tubes were monitored and weighed weekly on a balance (Denver, Germany; 210 g accuracy ± 0.1 mg) and calibrated for release over 8 weeks. The release rate was calculated by linear regression considering the difference in tube weights during the calibration period. The calibrated tubes were placed in the rumen 10 days before the commencement of daily methane measurements. The mean SF_6_ release rate from the permeation tubes in this study was 3.47 ± 0.46 mg/day. The PVC canister for the background sample was connected to the nylon and capillary tubes (Supelco, Cat#56712-U, ID 1/16) and Quick connectors (Swagelok, Cat#B-QC4-D-200) assembled by following the standard guidelines (Williams et al., 2014). The canister for the daily background sample was hung on the iron wire mesh fixed in the cemented wall on the east side. The vacuumized PVC canisters (> 95 kPa) were tied individually to collect breath samples for 24 h. Similar timings were maintained for tying and removal of the canisters throughout the measurement period. The post-collection canister pressure was measured by a digital pressure meter (Leo 2, Keller, Switzerland). Thereafter, the breath and background samples in the canister were diluted (2.15–3.0-fold) with high-purity N_2_ gas, and pressure was measured to calculate the dilution factor.

The successive subsamples were collected in a gas airtight glass syringe (Hamilton, 1 ml) for analyzing the methane and SF_6_ concentrations using a gas chromatograph (GC 2010 plus; Shimadzu, Japan). The GC was fitted with a FID and electron capture detector for the analysis of methane and SF_6_ concentrations, respectively. In brief, the following chromatographic conditions were maintained for the SF_6_ analysis: inlet temperature of 100°C, column temperature of 40°C, detector temperature of 250°C, airflow rate of 400 ml/min, hydrogen flow rate 40 ml/min, and nitrogen flow rate of 30 ml/min. In contrast, the following conditions were applied for the methane analysis: inlet temperature of 100°C, column temperature of 60°C, detector temperature of 150°C, airflow rate of 400 ml/min, hydrogen flow rate of 40 ml/min, and nitrogen flow rate of 30 ml/min. Methane (ppm) and SF_6_ (ppt) concentrations were calculated using the canister pressures at different time points (Lassey et al., 2014) with a slight modification for local elevation and atmospheric pressure:


$$\left[{\text{G}}_{\text{S}}\right]=\frac{90-\tau_{\text{f}}}{\tau_{\text{e}}-\tau_{\text{s}}}\times \left[{\text{G}}_{\text{A}}\right]$$

G_*S*_ represented the concentration of methane (ppm) or SF_6_ (ppt) at an atmospheric pressure of 90 kPa and elevation of 920 m. τ_*f*_ (kPa) was the final vacuum in the canister after N_2_ dilution, τ_*s*_ (kPa) is the post-sampling vacuum in the canister, τ_*e*_ is the vacuum in the evacuated canister, and G_*A*_ is the concentration of methane (ppm) or SF_6_ (ppt) in the samples presented to the GC.

Daily enteric methane emissions were calculated using the equation of Moate et al. (2014).


$${\text{R}}_{\mathrm{CH4}}={\text{R}}_{\mathrm{SF6}}\,\frac{{\left[{\text{CH}}_{4}\right]}_{\text{M}}-{\left[{\text{CH}}_{4}\right]}_{\text{BG}}}{{\left[{\text{SF}}_{6}\right]}_{\text{M}}-{\left[{\text{SF}}_{6}\right]}_{\text{BG}}}\times \frac{{\text{MW}}_{{\text{CH}}_{4}}}{{\text{MW}}_{{\text{SF}}_{6}}}\times 1,000$$

R_*CH4*_ is the CH_4_ emission (g/day), R_*SF6*_ is the SF_6_ release rate from the tubes (mg/day), [CH_4_]_*M*_–[CH_4_]_*BG*_ is the methane concentration (ppm) in the sample and background, [SF_6_]_*M*_–[SF_6_]_*BG*_ is the methane concentration (ppm) in the sample and background, and MW_*CH4*_ and MW_*SF6*_ represent the molecular mass of CH_4_ and SF_6_, respectively.

The DMI (g/day) along with daily methane emission (g/day) recorded over 10 days during methane measurement trials were used for the calculation of methane yield (MY, g/kg DMI). Daily methane emission (g/day) was divided by the mean DMI (g/day) over the measurement period. After 180 days, oil supplementation for the CON and INT groups was discontinued, and all the animals received a similar basal diet without SWPO. After 30 days of oil withdrawal, daily enteric methane emission was again quantified for 10 days along with the recording of DMI (g/day). The enteric methane emission was measured following the method described previously in this section.

### Ruminal Fluid Collection

The day that the methane measurements ended (day 180), rumen fluid samples were collected 3 h post-feeding from each sheep using a nylon stomach tube (length 1 m). The stomach tube and handheld vacuum pump (Mityvac 8,000; Lincoln Industrial, St. Louis, United States) were connected to a sample collection vessel. The first 30 ml of rumen fluid was discarded to avoid saliva contamination and then 45 ml of rumen fluid was collected and strained through a muslin cloth. The tubes containing strained ruminal fluid samples for DNA isolation and fermentation parameters (two sets of 15 ml each) were placed on ice for immediate transportation to the laboratory. Another set of tubes containing 15 ml of strained fluid were transported to the laboratory without placement in an icebox for protozoal enumeration. The first subset was centrifuged at 13,600 × *g* for 15 min, and after adding 2–3 drops of saturated HgCl_2_ and metaphosphoric acid (25%) in 1:4 (v/v), the supernatant was preserved for ammonia-N and VFA estimation, respectively. The second subset of strained fluid was used for protozoal enumeration on the same day, whereas the third subset of ruminal fluid was centrifuged at low speed (1,000 rpm, 5 min) and the supernatant was preserved at −80°C until used in the process of DNA isolation.

### Protozoal Enumeration

The protozoa in the rumen fluid were enumerated as per the method of Kamra and Agarwal (2003) using a Neubauer counting chamber. In brief, a 5-ml sample was pipetted into a screw cap tube containing an equal volume of 37% formaldehyde. Two drops of methyl green (70 μl) and glacial acetic acid (2 ml) diluted to 100 ml with distilled water were added to it and stored overnight at room temperature. The ruminal protozoa were enumerated in 30 microscopic fields. Ruminal protozoa enumeration and morphological identification were carried out under a phase-contrast microscope (Nikon Eclipse, Japan) at ×10 objective. The ruminal protozoa based on the morphology and cilia distribution were categorized as *Entodiniomorphs* and *Holotrichs* (Hungate, 1966). The protozoal numbers in the rumen fluid were calculated using the following formula:


$$\text{N}=\frac{\text{n}\times \text{A}\times \text{D}}{\text{a}\times \text{v}}$$

where N is the number of protozoa (cells) in 1 ml of rumen fluid, n is the average cell count per microscopic field, A is the area of the slide on which the diluted rumen fluid sample spread, D is the dilution of rumen fluid, a is the area of the microscopic field, and v is the volume of rumen fluid in the cavity. The protozoal numbers were expressed as × 10^7^ or × 10^6^ cells/ml.

Following the methane measurements in the oil discontinuation study, the rumen protozoa in each group were enumerated as per the method described previously.

### DNA Isolation

The samples were initially centrifuged at 13,400 × *g* and 4°C for 10 min, and the pellet was retained by discarding the supernatant. Thereafter, 1 ml of lysis buffer as described by Yu and Morrison (2004) was added to dissolve the pellet by gentle pipetting. The contents were transferred to a 2-ml sterile screw cap tube with an O-ring (BioSpec, United States) and contained 0.5 g (0.1 mm diameter) pre-sterilized zirconia beads (BioSpec, United States). The RBB+C method of Yu and Morrison (2004) was employed for genomic DNA isolation. Genomic DNA quality was checked using 0.8% agarose gel electrophoresis, while the DNA concentration was ascertained with Qubit 4.0 (Thermo Fisher Scientific, Waltham, United States).

### Library Preparation and Sequencing

Amplicon libraries were prepared using a Nextra XT kit (Illumina Inc., San Diego, United States). The archaea-specific primer sequences *Arch-344F* (Wemheuer et al., 2012) and *Arch-806R* (Takai and Horikoshi, 2000) were synthesized along with Illumina recommended adapters and error-correcting barcodes unique to each sample. The PCR amplification was performed as follows: initial denaturation at 95°C for 3 min followed by 25 cycles of denaturation at 95°C for 30 s, annealing at 55°C for 30 s, extension at 72°C for 30 s, and final extension at 72°C for 5 min. A reaction without template was used as a negative control, whereas the *Methanobrevibacter smithii* DNA template was used as a positive control during the PCR amplification. All 18 amplicon libraries were purified with AMPureXP beads (Beckman Coulter Life Sciences, United States) and analyzed individually on a 4,200 Tape Station (Agilent Technologies, United States). The libraries were multiplexed (10–20 pM of each) and sequenced on an Illumina MiSeq platform (Illumina, San Diego, CA, United States) using MiSeq reagent kit v3, and 2 × 300 bp paired-end reads were generated to obtain approximately 0.1 million sequences per library.

### Statistical Analysis

All the data were checked for a normal (Gaussian) distribution in GraphPad Prism version 9 (GraphPad Software, San Diego, United States) using the D’Agostino–Pearson normality test at the 0.05 alpha level. The data pertaining to daily methane emissions, nutrient digestibility, fermentation, and rumen protozoa were analyzed in SPSS version 21.0 (IBM SPSS, United States) using ANOVA with the following model.


$${\text{Y}}_{\text{ij}=}\,\mu +{\text{A}}_{\text{i}}+\varepsilon_{\text{ij}}$$

Y_*ij*_ represents individual observation, μ represents the population mean, A_*i*_ represents the treatment effect, and ε_*ij*_ represents experimental error. The difference among means was compared using Tukey’s *post hoc* method and considered significant at *p* ≤ 0.05. The impact of SWPO withdrawal and previous methane emissions were compared with paired *t*-tests in GraphPad Prism version 9. The correlation matrix between multiple variables was performed in GraphPad Prism version 9, and Pearson correlation coefficients (r) considering Gaussian distributions were calculated at the 95% CI.

### Bioinformatics Analysis

The amplicon sequences were processed using DADA2 v1.16 (Callahan et al., 2016) in R v4.0.2. The sequencing quality was assessed using the functions plotQualityProfile, dereplication, denoising, and merging. Chimeras were removed from the filtered reads using the function removeBimeraDenovo. Fasta files compatible with DADA2 for taxonomy assignments were generated using Rumen and Intestinal Methanogen-DB (Seedorf et al., 2014). Furthermore, the archaea-specific database RIM-DB was used for taxonomy classification, and the annotated taxonomy table from DADA2 was imported into phyloseq (McMurdie and Holmes, 2013) in R. The ASVs (amplicon sequence variants) with low abundance were pruned and rarefied to the lowest numbers (31,044 sequences) to examine the archaeal diversity measures. The rarefaction curve was plotted using the vegan package V2.0-7 (Oksanen et al., 2013), and the Shannon index and *post hoc* comparisons were performed using Wilcoxon rank sum tests. The multivariate homogeneity of group dispersion was tested using the betadisper function of the vegan package. The ASV count data were scaled to per 10,000 sequences, taxonomically annotated at various ranks, and then the relative abundance plots were generated using ggplot2 (Wickham, 2011). Differential abundance was calculated using DESeq2 and significance analysis was performed using the Wald parametric test with Benjamini–Hochberg correction (Love et al., 2014). The diet effect on the diversity of methanogens at different taxonomic ranks was studied across groups and tested for significance using PERMANOVA with 999 permutations. The core microbiome analysis was performed in microbiome V1.4.1 (Lahti and Shetty, 2012) in R by keeping a minimum prevalence and detection threshold of 50% and 0.01, respectively.

## Results

### Intake, Digestibility, and Average Daily Gain

The mean DMI in the CTR (884 g/day), CON (900 g/day), and INT (893 g/day) groups was similar (*p* = 0.15), and there was no adverse impact of the long-term (180 days) SWPO supplementation on the DMI in the sheep (Table 2). The mean DMI during withdrawal period in CTR (967 g/day), CON (1,014 g/day), and INT (988 g/day) groups was also similar (*p* = 0.176). The intake (g/day) of OM, CP, NDF, and ADF in the CON and INT groups did not differ from the CTR group. However, the intake of EE in the CON (36.2 g/day) and INT (36.0 g/day) groups was significantly higher (*p* < 0.01) than in the CTR group (18.0 g/day). Similarly, the ME intake in CON (11.0 MJ/day) and INT (10.7 MJ/day) groups was also significantly higher (*p* < 0.01) than in the CTR group (10.3 MJ/day). The apparent digestibility (%) of all the nutrients except EE in the CON and INT groups was comparable with the CTR group. Long-term feeding of SWPO led to a higher (*p* = 0.02) body weight in the CON group (33.0 kg) compared with the CTR group (29.9 kg). However, the difference in body weight between CTR and INT as well as CON and INT after 180 days of SWPO feeding was similar. A significant difference (*p* = 0.02) in the ADG between CON (49.3 g/day) and CTR (32.7 g/day) was recorded in this study. On the other hand, the difference in ADG (g/day) in sheep between the CTR-INT and CON-INT groups was non-significant. In the present study, a positive correlation (*r* = 0.53) between the ME intake (MJ/day) and ADG (g/day) was recorded. During the entire experimental period of 180 days, the animals consumed 3.24 and 1.61 L of SWPO in the CON and INT groups, respectively. The corresponding cost of the daily SWPO in the CON and INT groups was INR 0.72 and 0.36, respectively (1 US$ = 75 INR).

**TABLE 2:** Effect of silkworm pupae oil supplementation on intake and nutrient digestibility.

Attributes	CTR	CON	INT	SEM	*P*-value
Initial BW (kg)	24.1	24.1	24.1	0.273	0.996
Final BW (kg)	29.9^a^	33.0^b^	30.8^ab^	0.492	0.022
BW gain (kg)	5.87	8.88	6.63	0.552	0.058
ADG (g/day)	32.7^a^	49.3^b^	36.9^ab^	3.07	0.024
**Intake (g/day)**					
DM	884	900	893	3.42	0.155
OM	818	832	826	3.11	0.151
CP	96.1	96.7	96.5	0.13	0.157
NDF	507	518	513	2.29	0.152
ADF	295	304	300	1.74	0.153
EE	18.0^a^	36.2^b^	36.0^b^	0.83	<0.0001
Energy* (MJ/day)	10.3^a^	11.0^c^	10.7^b^	0.05	<0.0001
**Apparent digestibility (%)**					
DM	68.8	67.2	67.9	0.29	0.093
OM	69.7	68.4	68.7	0.28	0.127
CP	67.6	65.9	65.6	0.42	0.109
NDF	63.1	61.7	62.8	0.34	0.210
ADF	48.3	48.0	48.7	0.48	0.842
EE	78.6^a^	87.6^b^	88.0^b^	0.46	<0.0001
**Methane emission**					
Daily methane (g/day)	23.6^c^	17.5^a^	18.0^ab^	0.918	0.008
Methane yield (g/kg DMI)	26.7^c^	19.3^a^	20.3^ab^	0.101	0.004
Methane (g/day) during withdrawal period^#^	23.3	22.8	24.4	2.06	0.208

CTR, control; CON, daily oil supplementation; INT, biweekly oil supplementation; DM, dry matter; OM, organic matter; CP, crude protein (N_2_ × 6.25); NDF, neutral detergent fiber; ADF, acid detergent fiber; EE, ether extract. Mean values bearing different superscripts in a row differ significantly (p < 0.05). *Estimated using dry matter intake (g/day) and feed energy (ME MJ/kg). ^#^Measurement after 30 days of the withdrawal of oil supplementation.

### Methane Emissions

**FIGURE 1:**
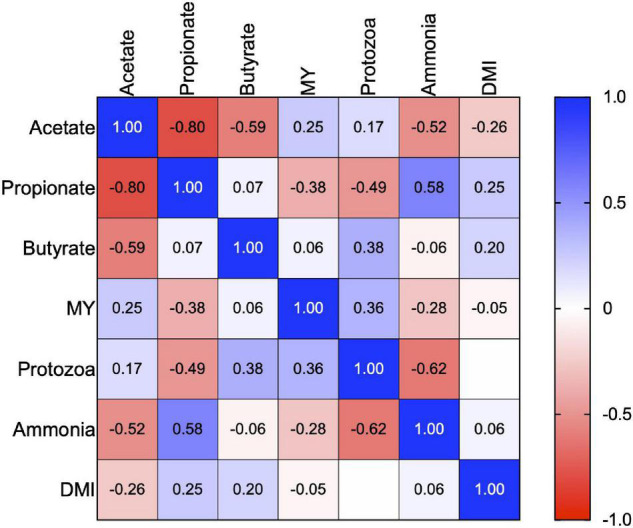
Correlation matrix (Pearson) between MY, ruminal protozoa, DMI, and individual volatile fatty acids. The scalar colors symbolize the positivity (closer to 1, blue squares) or negativity (closer to -1, red squares) of the correlation. MY is the methane yield, and DMI is dry matter intake in the correlation matrix.

Daily enteric methane emissions in the CON (17.5 g/day) and INT (18.0 g/day) groups were significantly lower (*p* < 0.01) than in the CTR group (23.6 g/day). Data on daily methane emissions (g/day) revealed a reduction of about 23–25% in the CON and INT groups due to the daily or biweekly supplementation of SWPO (Table 2). However, the daily enteric methane emission (g/day) between the CON and INT groups did not differ significantly (*p* = 0.008). Similarly, the difference in daily enteric methane emission (g/day) between the supplementation and non-supplementation days in the INT group was not significant (*p* = 0.77). A uniform comparison of methane emission (MY) among the groups also revealed a significant reduction (*p* < 0.01) in the MY in CON (19.3 g/kg DMI) and INT (20.3 g/kg DMI) groups compared with that of the CTR group (26.7 g/kg DMI); however, the MY (g/kg DMI) between the CON and INT groups was non-significant. There was a weak negative correlation (*r* = -0.05; *p* = 0.80) between the MY and DMI (Figure 1). MY was positively correlated with protozoa numbers (*r* = 0.36) and acetate (*r* = 0.25). The enteric methane emissions (g/day) among the sheep of different groups were similar (*p* = 0.208) during the withdrawal period (Table 2).

### Ammonia, Volatile Fatty Acids, and Metabolic Hydrogen

The ruminal ammonia concentration in the CON and INT groups compared with the CTR group (19.7 mg/100 ml) was significantly lower (*p* < 0.01). Similarly, the ammonia concentration was also different (*p* < 0.01) between the CON and INT groups. On the other hand, total volatile fatty acid (TVFA) production was similar (*p* = 0.99). Despite the similar TVFA concentration, the propionate production with reference to CTR (11.3 mmol) was higher (*p* < 0.01) in the SWPO supplemented CON (13.0 mmol) and INT (12.3 mmol) groups (Table 3). However, variation in the concentration of other VFA between the groups was non-significant. The A/P ratio in the CON and INT groups was significantly lower (*p* < 0.01) than that in the CTR group.

**TABLE 3:** Effect of oil supplementation on the rumen fermentation and protozoan numbers.

Attributes	CTR	CON	INT	SEM	*P*-value
NH_3_-N (mg/100 ml)	19.7^c^	18.8^b^	17.9^a^	0.20	<0.001
Total VFA (mM)	68.5	68.7	68.4	1.29	0.997
**VFA (mmol)**					
Acetate	51.9	50.5	50.7	1.03	0.850
Propionate	11.3^a^	13.0^b^	12.3^b^	0.25	0.006
Iso-butyrate	0.75	0.63	0.68	0.04	0.516
Butyrate	3.49	3.51	3.58	0.17	0.977
Iso-valerate	0.82	0.77	0.80	0.04	0.900
Valerate	0.24	0.23	0.27	0.01	0.077
A/P ratio	4.59^c^	3.87^a^	4.12^b^	0.08	<0.001
**Ruminal protozoa**					
Total (×10^7^ cells/ml)	9.26^c^	6.51^b^	5.30^a^	0.282	<0.001
*Entodiniomorphs* (×10^7^ cells/ml)	8.87^c^	6.25^b^	5.08^a^	0.275	<0.001
*Holotrichs* (×10^6^ cells/ml)	3.87^b^	2.54^a^	2.19^a^	0.118	<0.001
**Withdrawal**					
Total (×10^7^ cells/ml)	9.21	8.62	8.69	0.130	0.113
*Entodiniomorphs* (×10^7^ cells/ml)	9.01	8.40	8.48	0.123	0.092
*Holotrichs* (×10^6^ cells/ml)	0.73	0.78	0.75	0.031	0.743

*CTR, control (no oil supplementation); CON, daily oil feeding; INT, biweekly oil feeding; NH_3_-N, ammonia nitrogen; VFA, volatile fatty acid. Mean values bearing different superscripts in the same row differ significantly (p < 0.05).*

In the CTR, CON, and INT groups of the present study, the production of metabolic hydrogen was 130, 128.5, and 128.6 mmol; however, the utilization of produced metabolic hydrogen in methanogenesis was 73, 54, and 56% in the respective groups. Results from the study indicated that 16–18% less metabolic hydrogen was utilized in CON and INT groups on account of reduced methanogenesis.

### Rumen Protozoa

Supplementation of SWPO lowered (*p* < 0.01) the numbers of total protozoa (×10^7^ cells/ml) in the CON (6.51) and INT (5.30) groups compared with the CTR group (9.21). Similarly, the numbers of *Entodiniomorphs* (×10^7^ cells/ml) and *Holotrichs* (×10^6^ cells/ml) in the CON and INT groups relative to the CTR group were low (*p* < 0.01) (Table 3). Moreover, the difference in the total protozoa, *Entodiniomorphs*, and *Holotrichs* between the CON and INT groups was also significant (*p* < 0.01). The ammonia and ruminal protozoa (*r* = −0.62), and propionate and protozoa (*r* = −0.49) negatively correlated (Figure 1). Data from the withdrawal study indicated similar numbers of total protozoa (*p* = 0.11), *Entodiniomorphs* (*p* = 0.09), and *Holotrichs* (*p* = 0.74) among the groups.

### Archaeal Diversity

**FIGURE 2:**
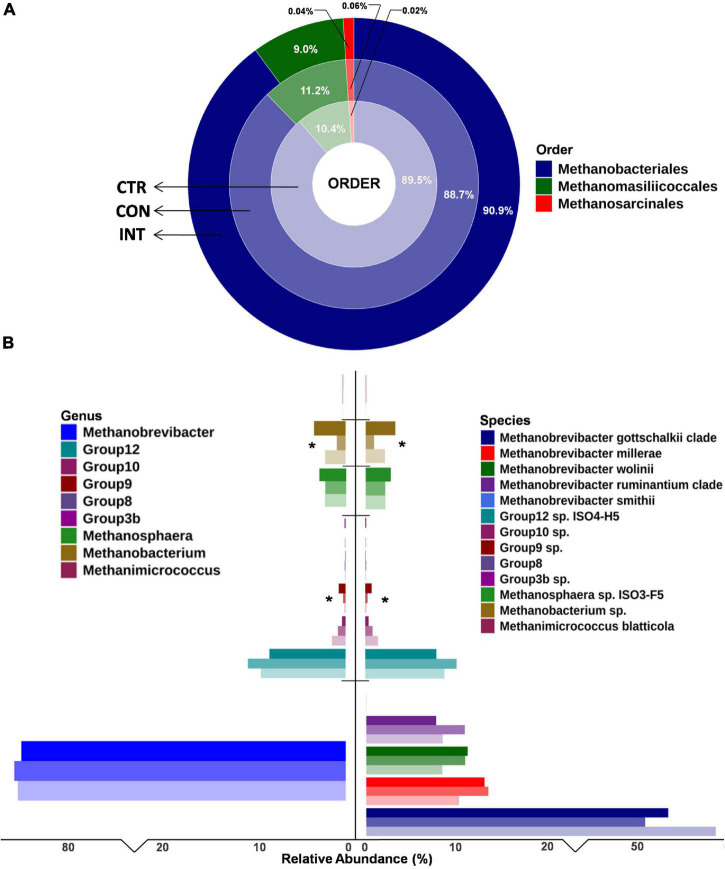
Ruminal archaea diversity in sheep from three different groups at **(A)** order level and **(B)** genus and species level. The increasing intensity of the same color shows the abundance in the CTR, CON, and INT groups. For instance, light blue indicates CTR, medium blue indicates CON, and dark blue indicates the INT group. *Denotes the significance in abundance among groups. CTR, control group (no oil); CON, daily oil supplementation; INT, biweekly oil supplementation.

Overall, 7,406,185 paired-end raw reads (mean 411,454) per sample were generated in this study. After quality filtering and chimeric removal, a total of 4,735,849 paired-end reads (263,103 ± 38,445) were retained for further analysis comparing ruminal archaea diversity. The filtered reads were categorized into 69 archaeal ASVs (Supplementary Figure 1). At the order level, the *Methanobacteriales* represented the most significant fraction of the ruminal archaea; however, no significant differences in abundance of archaea at the order level between the CON, CTR, and INT groups was observed (Figure 2 and Supplementary File 1). Similarly, methanogens in this study were affiliated to three families, namely *Methanobacteriaceae*, *Methanomassilliicoccaceae*, and *Methanosarcinaceae*. *Methanobacteriaceae* constituted 89.8% (range 88.6–91.0%) of the total rumen archaea and their distribution remains unaffected with the oil supplementation.

**FIGURE 3:**
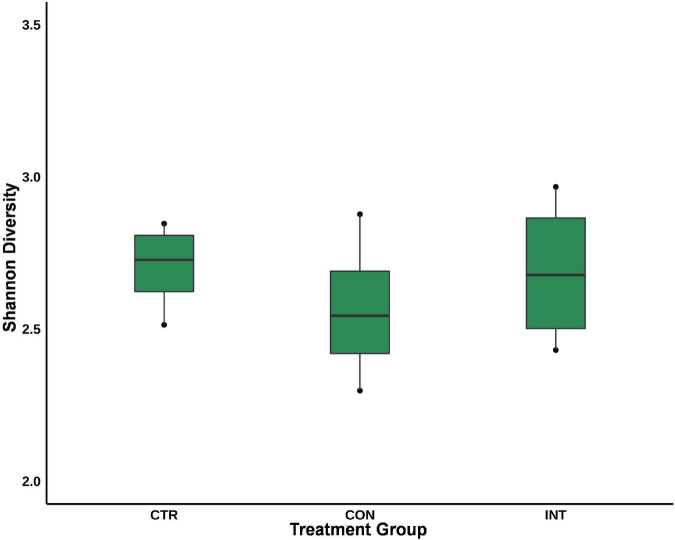
Alpha diversity of the ruminal archaea in sheep supplemented with silkworm pupae oil. CTR, control group (no oil); CON, daily oil supplementation; INT, biweekly oil supplementation.

Similarly, at the genus level, *Methanobrevibacter* was the most prominent genera in the sheep rumen; however, their proportion was not significantly different (*p* = 0.98) between the three groups. Despite their limited proportions, the abundance of *Group9* (*p* = 0.04) and *Methanobacterium* (*p* = 0.04) was significantly different among the groups. The *group9* archaea were less abundant in the CTR group (0.12%) compared with the CON (0.26%) and INT (0.71%) groups, whereas the *Methanobacterium* proportion in the CON group (0.91%) was significantly lower (*p* = 0.04) than in the CTR (2.10%) and INT (3.24%) groups. At the species level, the proportions of *Group*3*b* (*p* = 0.06), *Group*9 (*p* = 0.03), and *Methanobacterium* (*p* = 0.04) were different between the groups (Figure 2). The results for the archaeal proportion in the present study indicated that ∼98% of the ruminal archaea remain unaffected by the oil supplementation, and only a minor fraction of the archaea (< 2%) showed a proportional increase or decrease (Figure 2). Moreover, the Shannon index, which estimates alpha diversity, did not reveal any significant differences in the archaeal diversity between the groups (Figure 3).

### Core Archaeome

**FIGURE 4:**
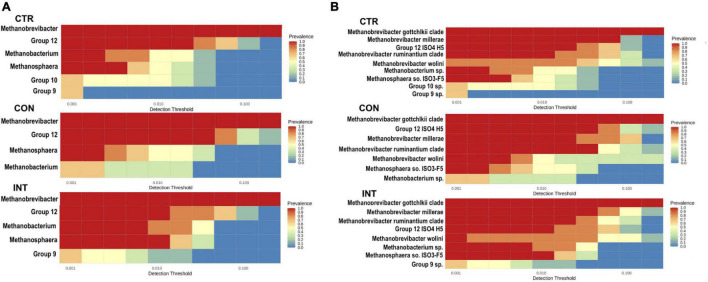
Ruminal archaea representing the core microbiome at a minimum prevalence of 50%. **(A)** Represents the core microbiome at the genus level in the CTR, CON, and INT groups, while **(B)** represents the core microbiome at the species level in the respective groups. The color gradients indicate the variability in prevalence. CTR, control; CON, daily oil supplementation; INT, biweekly oil supplementation.

At a minimum prevalence of 50% and a minimum detection threshold of 0.01, each group’s core archaeome was computed at the genus (Figure 4A) and species levels (Figure 4B). *Methanobrevibacter*, *Group12*, *Methanobacterium*, and *Methanosphaera* constituted the core archaeome across the groups. However, *Group9* was exclusively present in the CTR and INT groups, whereas *Group10* was present only in the CTR group. Comparison of archaeome at the species level revealed the presence of the *Methanobrevibacter gottschalkii clade*, *Group12* sp. *ISO4-H5*, *Methanobrevibacter millerae*, the *Methanobrevibacter ruminantium clade*, *Methanobrevibacter wolinii*, *Methanosphaera IS03-F5*, and *Methanobacterium* sp. across the groups. The differences were observed in the proportion of *Group10* sp. and *Group9* sp. These two species were exclusively absent in the CON group. This analysis revealed that the core archaeome in the CON group was distinct from the CTR and INT groups.

## Discussion

SWPO supplementation resulted in additional body weight gains of 3.1 and 0.9 kg in the CON and INT groups, respectively, as compared with the CTR group. Supplementation of SWPO in CON group led to an additional ADG (16.6 g/day). The lower body weight (kg) and ADG (g/day) in our study were mainly due to the maturity of the experimental animals (18 months). Body weight gain and ADG would have been higher if the animals were growing (i.e., at the age of 3–6 months).

Lipid supplementation is one of the established methods for reducing enteric methane emission (Pinares-Patińo et al., 2016). The study also established that the SWPO supplementation in daily (CON) or biweekly (INT) regimes was equally effective in reducing methane emissions. Methane yields (g/kg DMI) reported here were consistent with previous reports (Pinares-Patino et al., 2014; Charmley et al., 2016; Williams et al., 2019). The reduction in daily methane emissions (g/day) due to long-term supplementation of SWPO was 5–8% higher than a previous short-term study (Thirumalaisamy et al., 2020). The supplementation of lipids mitigate methane emission via inhibition of hydrogen-producing microbes (Tapio et al., 2017), impeding microbial colonization and increasing hydrogen sequestration (van Nevel and Demeyer, 1992) and a shift in microbiota (Vargas et al., 2020). However, the extent of methane reduction depends on the source of the lipids and the fatty acid composition. A significant reduction (23–26%) in daily enteric methane emissions due to SWPO supplementation demonstrated its anti-methanogenic effectiveness. A recent study (Jayanegara et al., 2020) reported that supplementation with 5% insect oils effectively reduced *in vitro* methane production. Like cricket and mealworm oil (Jayanegara et al., 2020), the SWPO also possesses a relatively higher proportion of unsaturated fatty acids (65.5%) than saturated. Oleic (C18:1) and linolenic (C18:3) are two primary unsaturated fatty acids in SWPO that aggregately represent 58.4% of the total fatty acids (Thirumalaisamy et al., 2020). One plausible explanation for the reduction in methane emission can be the high degree of fatty acid unsaturation.

The negative correlation between DMI (g/day) and MY (g/kg DMI) is consistent with previous studies (Muetzel, 2011; Hristov and Melgar, 2020). In addition, the higher energy intake (ME MJ/day) in test groups due to oil supplementation could be one of the reasons for the reduction in methane emissions. However, Bayat et al. (2018) did not observe any difference in MY due to the variable energy density.

Total VFA and acetate production were unaffected by the SWPO supplementation. However, a shift in the fermentation pattern with more propionate and a decreased acetate to propionate ratio was apparent in this study. The higher propionate production and lower acetate to propionate ratio without any apparent change in total VFA and acetate concurs with the findings of previous studies (Jalè et al., 2006; Vargas et al., 2020). The reduction in rumen methanogenesis in the CON and INT groups could be due to the additional propionate production, which utilized spared H_2_. Fat supplementation is believed to be toxic to the rumen protozoa (Machmüller et al., 1998); however, the negative impact is not uniform across fat/oil sources and varies with the degree of unsaturation (Jenkins, 1993). The negative impact of SWPO supplementation on protozoa was in this study can be attributed to the high degree of unsaturation in the oil (Thirumalaisamy et al., 2020). It is well established that defaunation leads to the proliferation of succinate-producing bacteria (Kurihara et al., 1978; Ungerfeld et al., 2020), resulting in more propionate production (Eugène et al., 2004; Li et al., 2018). This was also substantiated by our results demonstrating a negative correlation between rumen protozoa and propionate production.

After methanogenesis, propionate is considered another major sink for H_2_ removal (Janssen, 2010). The H_2_ spared from reduced methanogenesis was, however, not completely redirected to propionate production, indicating that a major portion of H_2_ remains unaccounted for. These findings are consistent with previous reports (Anderson et al., 2010; Ungerfeld, 2015), implying that caproate and formate may be two other major sinks for H_2_ utilization.

Rumen protozoa are not essential for animal survival, but due to their functional association with methanogens (Newbold et al., 2015), they held accountable for nearly 37% of methane emissions (Machmüller et al., 2003). Results indicated that the SWPO supplementation effectively reduced the protozoal numbers with a concurrent decrease in enteric methane emission. After the withdrawal of the oil supplementation, an increase in both the protozoal numbers and methane emission confirmed their involvement in methane emission. This is consistent with a previous report (Sutton et al., 1983) confirming that the discontinuation feeding with oils increased protozoa numbers to the pre-supplementation level. The role of rumen protozoa in bacterial predation is well known (Williams and Coleman, 1992) and *Entodiniomorphs* are more efficient predators than are the *Holotrichs* (Belanche et al., 2012). Although we have not explored the bacterial community in the present study, a shift in the bacterial community due to the reduction in predation by protozoa is expected. In a meta-analysis, Newbold et al. (2015) reported a 9% increase in the bacterial populations due to defaunation. One of the most consistent consequences of defaunation is the reduction in ammonia concentration (Newbold et al., 2015), which is also evident in the current study. This could be explained by the reduced bacterial breakdown due to the lower protozoal numbers (Williams and Coleman, 1992); however, it needs further confirmation.

There was little impact of protozoal reduction on the methanogens community. This contradicts a previous study (Tan et al., 2020) that reported a significant effect of protozoa reduction on the methanogen community. In the current study, *Methanobrevibacter*, despite being the prominent archaeal genus, was similarly distributed between test (CON and INT) and CTR groups. This is consistent with an earlier short-term study (Thirumalaisamy et al., 2020) that evaluated SWPO at a similar level. Similarly, the distribution of the prominent species *Methanobrevibacter gottschalkii* was also uniform among the groups. These findings indicate that the core methanogens in sheep remain unaffected by the daily or biweekly supplementation of SWPO. A previous study (St-Pierre et al., 2015) also confirmed the dominance of *Methanobrevibacter* in the sheep rumen. Only minor methanogens such as *Group3b*, *Group9*, and *Methanobacterium* having a distribution frequency of < 2% in the archaeal community were affected with the SWPO supplementation. *Group3b* methanogen was recently identified in the rumen (Jin et al., 2017) but with a limited distribution of ∼0.2%. The contribution of *Group3b* to rumen methanogenesis is yet to be investigated.

SWPO is readily accessible in India (Srinivas et al., 2019) and cheaper than conventional edible oil sources (Government of India, 2021). Animals in the CON and INT groups consumed 3.24 and 1.61 L of oil during the experimental period of 180 days. The daily financial cost (INR) for feeding SWPO in the CON and INT groups was only 0.72 and 0.36, respectively (1 US$ = 75 INR). Based on the prevailing price of mutton (INR 600/kg), sheep in the CON group, due to the SWPO supplementation, had an additional ADG equivalent of INR 9.96 per day. However, the supplementation in the INT group led to an additional ADG of 4.2 g—only INR 2.52 per day. Thus, it would be far more economical for the farmers to feed SWPO daily (CON) at the recommended level of 2%. Although we attempted the SWPO supplementation through automatic oral drenching in the present study, it may not be practicable in a farm setting. Adding SWPO to concentrate mixture would be the most practical option on a farm, which requires further investigation.

## Conclusion

From the present study, it can be concluded that the supplementation of SWPO as 2% of DMI over the long term, with daily or biweekly supplementing, decreased daily methane emissions of sheep by 23–26%. The supplementation of SWPO, either through daily or biweekly regimes, was equally effective in reducing methane emissions; however, the daily supplementation of SWPO at the recommended level led to a significant increase in body weight gain and average daily gain compared with animals in the control and biweekly groups. It can be inferred from the discontinuation study that the daily supplementation of SWPO is required to achieve a persistent reduction in enteric methane emission, and withdrawal of the oil supplementation led to an increase in enteric methane emission to the pre-supplementation level. The oil did not significantly change the archaeal community composition but persistently decreased the ruminal protozoa numbers. The feeding of SWPO may lead to a relatively higher average daily gain in growing sheep and, therefore, could result in a net profit. Drenching of oil may not be practically feasible in the farm settings; therefore, further studies will be required to investigate the methane mitigation potential of including SWPO into formulated concentrates.

## Data Availability Statement

The datasets presented in this study can be found in online repositories. The names of the repository/repositories and accession number(s) can be found in the article/Supplementary Material.

## Ethics Statement

The animal study was reviewed and approved by Institute Animal Ethics Committee, National Institute of Animal Nutrition and Physiology.

## Author Contributions

PM, AK, and RB conceived and designed the study. GT performed the methane measurement, digestibility studies, executed the chemical composition, and ruminal fluid analysis. ST and AK performed the molecular bioinformatics analysis, and visualization of the data. All authors participated in the data analysis and article writing.

## Conflict of Interest

The authors declare that the research was conducted in the absence of any commercial or financial relationships that could be construed as a potential conflict of interest.

## Publisher’s Note

All claims expressed in this article are solely those of the authors and do not necessarily represent those of their affiliated organizations, or those of the publisher, the editors and the reviewers. Any product that may be evaluated in this article, or claim that may be made by its manufacturer, is not guaranteed or endorsed by the publisher.

## Supplementary Material

Supplementary Figure 1 Rarefaction plot (**A**, before; **B**, after) showing the archaeal richness in different groups of sheep receiving feed with or without silkworm pupae oil.

Supplementary File 1 Read stats, OTUs taxonomy and percent abundance.
