# Supplementary figures and images for: Effect of Long-Term Supplementation With Silkworm Pupae Oil on the Methane Yield, Ruminal Protozoa, and Archaea Community in Sheep
Source: Front Microbiol. 2022 Mar 8;13:780073. doi: 10.3389/fmicb.2022.780073 (PMC8964344; doi:10.3389/fmicb.2022.780073)

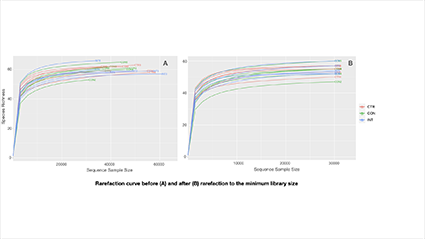

Supplement: Supplementary Figure 1 — Rarefaction plot (A, before; B, after) showing the archaeal richness in different groups of sheep receiving feed with or without silkworm pupae oil. [file Image_1.TIFF]
